# Impact of Wines and Wine Constituents on Cyclooxygenase-1, Cyclooxygenase-2, and 5-Lipoxygenase Catalytic Activity

**DOI:** 10.1155/2014/178931

**Published:** 2014-05-29

**Authors:** Zsofia Kutil, Veronika Temml, David Maghradze, Marie Pribylova, Marcela Dvorakova, Daniela Schuster, Tomas Vanek, Premysl Landa

**Affiliations:** ^1^Laboratory of Plant Biotechnologies, Institute of Experimental Botany AS CR, Rozvojova 263, 165 02 Prague 6-Lysolaje, Czech Republic; ^2^Department of Crop Sciences and Agroforestry, Faculty of Tropical AgriSciences, Czech University of Life Sciences Prague, Kamycka 129, 165 21 Prague 6-Suchdol, Czech Republic; ^3^Computer-Aided Molecular Design Group, Institute of Pharmacy/Pharmaceutical Chemistry and Center for Molecular Biosciences Innsbruck (CMBI), Innrain 80/82, 6020 Innsbruck, Austria; ^4^Institute of Horticulture, Viticulture and Oenology, Agricultural University of Georgia, David Aghmashenebeli Alley13-th Km, 0159 Tbilisi, Georgia

## Abstract

Cyclooxygenases and lipoxygenases are proinflammatory enzymes; the former affects platelet aggregation, vasoconstriction, vasodilatation and later the development of atherosclerosis. Red wines from Georgia and central and western Europe inhibited cyclooxygenase-1 (COX-1) activity in the range of 63–94%, cyclooxygenase-2 (COX-2) activity in the range of 20–44% (tested at a concentration of 5 mL/L), and 5-lipoxygenase (5-LOX) activity in the range of 72–84% (at a concentration of 18.87 mL/L). White wines inhibited 5-LOX in the range of 41–68% at a concentration of 18.87 mL/L and did not inhibit COX-1 and COX-2. Piceatannol (IC_50_ = 0.76 **μ**M) was identified as a strong inhibitor of 5-LOX followed by luteolin (IC_50_ = 2.25 **μ**M), quercetin (IC_50_ = 3.29 **μ**M), and myricetin (IC_50_ = 4.02 **μ**M). *trans*-Resveratrol was identified as an inhibitor of COX-1 (IC_50_ = 2.27 **μ**M) and COX-2 (IC_50_ = 3.40 **μ**M). Red wine as a complex mixture is a powerful inhibitor of COX-1, COX-2, and 5-LOX, the enzymes involved in eicosanoid biosynthetic pathway.

## 1. Introduction

Moderate consumption of wine is associated with reduced incidence of coronary heart diseases [1, 2]. Alcohol present in wine decreases platelet aggregation, resulting in reduced adherence to the endothelial surface of the arteries, blood coagulation, and thrombus formation [3, 4]. Besides alcohol, platelet aggregation could be decreased by the inhibition of cyclooxygenase-1 (COX-1) activity by phenolic compounds present in wine, such as resveratrol [5]. COX-1 catalyzes biosyntheses of thromboxanes, eicosanoids propagating platelet aggregation and vasoconstriction [6, 7]. Therefore, the inhibition of COX-1 (e.g., by aspirin which is a COX-1 selective inhibitor) is proposed for the prevention of cardiovascular diseases [8, 9]. On the other hand, the inhibition of the second cyclooxygenase isoform (COX-2) results in reduced production of prostacyclin which is a vasodilitator and antiaggregatory prostanoid. Therefore, the selective COX-2 inhibitors (coxibs) used as anti-inflammatory drugs increase the risk of heart incidents [10]. The second important biosynthetic pathway leading to the production of eicosanoids is mediated by 5-lipoxygenase (5-LOX). The final product of the 5-LOX pathway, leukotriene B_4_ (LTB_4_), is a mediator of several inflammatory diseases including atherosclerosis [11]. There are also wine constituents such as quercetin which are able to inhibit 5-LOX activity [12]. In contrast to the reports on positive effects of phenolic compounds, two recent studies describe strong activation of COX-1 and COX-2 catalytic activity by myricetin and quercetin indicating that the wine constituents could also increase production of eicosanoids [13, 14]. In the light of the mentioned reports, we decided to test wine as a complex mixture of various compounds for its inhibitory potential towards COX-1, COX-2, and 5-LOX. We compared the activity of Georgian red and white wines with wines produced in central and western Europe. In addition, we evaluated the inhibitory activity of 33 phenolic compounds commonly occurring in wine with the aim to determine the contribution of each compound to the overall effect of wine. Finally,* in silico* docking experiments were used to propose a binding mode of the most active compounds.

## 2. Materials and Methods

### 2.1. Standards and Chemicals

The tested compounds were purchased from Sigma-Aldrich, Czech Republic (anisic acid, apigenin, caffeic acid, catechin, cinnamic acid, coumaric acid, cyanidin-chlorid, delphinidin-chlorid, 3,4-dihydroxybenzoic acid, epicatechin, ferulic acid, gallic acid, kaempferol, luteolin,* m*-hydroxybenzoic acid,* p*-hydroxybenzoic acid, piceatannol, quercetin-dihydro, resveratrol, salicylic acid, syringic acid, tyrosol, luteolin-7-glucosid, myricetin, naringenin, sinapinic acid, and vanillic acid); HWI Analytik, Germany (chlorogenic acid); Roth, Germany (rutin); and Polyphenols Laboratories AS, Norway (delphinidin 3-O-**β**-glucopyranoside, malvinidin 3-O-**β**-glucopyranoside, peonidin 3-O-**β**-glucopyranoside, and petunidin 3-O-**β**-glucopyranoside).

Eicosatetraynoic acid (ETYA), ethanol (EtOH), calciumionophor A23187, dimethylsulfoxide (DMSO), arachidonic acid (AA), indomethacin, trypan blue, gentian violet, porcine hematin, L-epinephrine, Na_2_EDTA, formic acid, COX-1 from ram seminal vesicles, and human recombinant COX-2 were purchased from Sigma-Aldrich (Czech Republic). Dextran T-500 was purchased from Roth (Germany). Ammonium chloride (NH_4_Cl), disodium hydrogen phosphate (Na_2_HPO_4_), sodium chloride (NaCl), and potassium dihydrogen phosphate (KH_2_PO_4_) were obtained from Lachner s.r.o. (Czech Republic) and zileuton was donated by Farmak a.s. (Czech Republic). Potassium chloride (KCl) and sodium hydroxide (NaOH) were purchased from Lachema a.s. (Czech Republic). Tris was purchased from Bio-Rad (Czech Republic). Calcium chloride (CaCl_2_·2H_2_O) and acetic acid (CH_3_COOH) were obtained from Penta (Czech Republic).

### 2.2. Wine Samples

Samples of commercial wines from different regions of Georgia, Czech Republic, France, Italy, and Austria were provided by local producers or purchased from supermarkets or wine stores. A total of 26 red wines of the varieties Pinot Noir (*n* = 5), Cabernet Sauvignon (*n* = 7), Cabernet Moravia (*n* = 2), Seperavi (*n* = 9), cuvée of Saperavi and Saperavi Budeshuriseburi (*n* = 2), and Alexandrouli (*n* = 1) and 13 white wine samples of the varieties Chardonnay (*n* = 6), Sauvignon Blanc (*n* = 3), Rkatsiteli (*n* = 2), and cuvée of Rkatsiteli and other local varieties (*n* = 2) were assayed. Detailed information about the tested wines is included in Supplementary Table 1 (see Supplementary Material available online at http://dx.doi.org/10.1155/2014/178931). After a preliminary screening, the red wines were diluted in ratio 1 : 9 in water to reach the final concentration of 5 mL/L and white wines were tested undiluted resulting in a concentration of 50 mL/L for the COX-1 and COX-2 assays. Undiluted red and white wine samples were used in the 5-LOX assay resulting in a final concentration of 18.87 mL/L.

### 2.3. COX-1 and COX-2 Assays

The assay was performed according to the procedure previously described by Reininger and Bauer [15] with COX-1 from ram seminal vesicles and human recombinant COX-2. COX-1 (1 unit/reaction) or COX-2 (0.5 unit/reaction) was added to 180 *μ*L of incubation mixture that consisted of 100 mM tris buffer (pH 8.0), 5 *μ*M porcine hematin, 18 mM L-epinephrine, and 50 *μ*M Na_2_EDTA. The wine sample, tested compound diluted in DMSO, 12% ethanol (in case of blanks for the wine samples), or pure DMSO (in case of blanks for purified constituents) was added (10 *μ*L) and the mixture was preincubated for 5 min at room temperature. The addition of 5 *μ*L of 10 *μ*M AA started the reaction. After 20 minutes of incubation at 37°C, the reaction was stopped by 10 *μ*L of 10% formic acid. All samples were diluted 1 : 15 in ELISA buffer and the concentration of (prostaglandin E_2_) PGE_2_ produced by the reaction was determined by a PGE_2_ ELISA kit (Enzo Life Sciences, US) according to the manufacturer's instructions. Absorbance relative to PGE_2_ concentration was measured with a microplate reader Tecan Infinite M200 (Tecan Group, Switzerland) at 405 nm. The results were expressed as percentage inhibition of PGE_2_ formation against untreated samples (blanks).

### 2.4. 5-LOX Assay

The assay was perfomed in a slightly modified version of the standard method described previously [16]. Buffy coat (50 mL) obtained from healthy donors was sedimented in 20 mL of dextran solution (6% dextran T-500, 1% NaCl) at 4°C. After one hour, the supernatant was collected and centrifuged at 1600 rpm at 4°C for 10 min and then the supernatant was discarded. The obtained pellet was washed with phosphate buffered saline (PBS, 0.02% KCl, 0.024% KH_2_PO4, 0.8% NaCl, 0.288% Na_2_HPO_4_·12H_2_O, pH 7.4) and again centrifuged. The hereby obtained pellet was lysed (0.17% NH_4_Cl, 0.2% Tris, pH 7.2) for 5 min at room temperature and then centrifuged at 1400 rpm at 4°C for 5 min. The pellet was washed by PBS again and centrifuged at 1400 rpm at 4°C for 15 min. Finally, the pellet was dissolved in 3 mL of PBS and the cells were tested for the viability. The cells were diluted to the final concentration of 4500 cell/*μ*L.

The incubation mixture consisted of 225 *μ*L of the cell suspension, 10 *μ*L of 2 mM CaCl_2_, 10 *μ*L of 10 *μ*M ETYA, 5 *μ*L of tested sample (wine, compound dissolved in DMSO, 12% ethanol or pure DMSO in case of blanks), 10 *μ*L of 21 *μ*M calcium ionophor A23187, and 5 *μ*L of 120 *μ*M AA. The reaction was stopped after 10 min incubation at 37°C with 20 *μ*L of 10% formic acid. Samples were diluted 40 times in ELISA buffer and the concentration of LTB_4_ was measured using a commercial LTB_4_ ELISA kit (Enzo Life Sciences, US) according to the manufacturer's instructions. Absorbance relative to LTB_4_ concentration was measured at 405 nm using a Tecan Infinite M200. The results were expressed as percentage inhibition of LTB_4_ formation against untreated samples (blanks).

### 2.5. Docking

During the docking simulation, 3D conformers of the molecules were placed within the binding pocket of 5-LOX, generating a set of energetically favorable poses. These poses were then ranked according to a score that the docking program assigns to each pose, estimating the binding free energy. The best-ranked pose of each molecule was then further optimized and a 3D representation of its interaction pattern was calculated to analyze the structure-activity relationship.

The possible direct interactions of the active compounds with 5-LOX were simulated, and 3D geometries of the compounds were calculated with Omega 2.2.1. [17]. The docking simulation was performed with the software package GOLD 5.1 (GOLD, UK) using the X-ray crystal structure of 5-LOX from the Protein Data Bank (PDB, code: 3o8y [18]). The A-chain binding site was chosen for the docking. The structure of 5-LOX does not contain a cocrystallized ligand, so the binding site was defined in a 6 Å radius around the catalytic iron (Fe2_1_A). Water molecules inside the binding pocket were set on toggle and spin, which means that the program can either use them as binding partners in the binding site or disregard them if they provoke steric hindrance of ligand binding.

Scoring was performed with the GoldScore scoring function. For structure-activity analysis, the best-ranked resulting docking pose of each molecule was energetically minimized within LigandScout using the Merck Molecular Force Field 94 force field. 3D protein-ligand interaction patterns were generated in LigandScout 3.1. [19] using default settings.

### 2.6. Statistical Analysis

The COX-1, COX-2, and 5-LOX tests were performed in three independent experiments with two replicates. At least three concentrations were used for the calculation of IC_50_ values of the wine compounds. The inhibition of enzyme activity by wine samples is presented as mean values. IC_50_ values are presented as mean values ± standard error (standard deviation, SD) of the mean.

## 3. Results and Discussion

### 3.1. Inhibition of COX-1 and COX-2 by Wine

Red wines tested at the concentration of 5 mL/L showed considerable potential to inhibit COX-1 as well as COX-2 with the efficiencies presented in Table 1. On the other hand, white wines tested at a 10 times higher concentration (50 mL/L) were practically inactive (Table 2). The exceptions were two Georgian samples, Rkatsiteli (sample no. 37), and cuveé of Rkatsiteli + Mtsvane + Kakhuri + Khikhvi + Kisi (sample no. 38), reducing COX-1 and COX-2 activity around 95% and 65%, respectively (Table 2). Results expressed in Tables 1 and 2 also demonstrate that red wines preferentially inhibited COX-1 rather than COX-2 with ratios ranging from 2.1 to 3.7. COX-1 selectivity ratio for aspirin varies between different authors from 1.7 to 42 [20, 21] in cell-free assays. However, the ratios recorded for wine fall within the mentioned range. These results support the hypothesis that wine may act, similar to aspirin, via inhibition of COX-1, in decreased risk of thrombosis. The incidence of cardiovascular diseases (CVD) is also directly influenced by alcohol consumption. While low quantities of ethanol (20–30 g per day) decrease CVD incidence, the overconsumption results in an increased risk [2, 22, 23]. Therefore, the cardioprotective effect of red wine (in moderate doses) could also be explained by the effect of ethanol in combination with preferential inhibition of COX-1 activity.

Comparison of different red wine varieties showed only minor differences. Larger variation in inhibitory activity was observed among the individual samples. For example, semisweet Saperavi (sample no. 19) reduced COX-1 activity only by 38% in comparison to the average 84% of all Saperavi samples. High COX-1 and COX-2 inhibitory activity of two Georgian white wine samples (no. 37 and 38) could be explained by different (Kakhetian) technology used during fermentation (six months fermentation with pomace). It is known that the presence of grape skins, bunch stems, and seeds results in a higher content of phenolic compounds in wine [24].

These results indicate that the processing method influenced inhibitory activity more than the variety of wine or its geographical origin.

### 3.2. Inhibition of 5-LOX by Wine

Red and white wines were tested for inhibition of 5-LOX at a concentration of 18.87 mL/L (5 *μ*L of undiluted wine in 265 *μ*L of reaction mixture). As with COX inhibition, red wines (Table 1) were stronger inhibitors of 5-LOX than white wines (Table 2), although the difference between red and white was not as pronounced. Red wines which were weak inhibitors of cyclooxygenases (e.g., Cabernet Moravia) were strong 5-LOX inhibitors and vice versa the strong COX inhibitors (cuvée of Saperavi + Saperavi Budeshuriseburi wine) were weak 5-LOX inhibitors. These results indicate that inhibition of COX-1 and COX-2 is influenced by different compounds than the inhibition of 5-LOX or that the same compounds have different effects in on the respective enzymes.

### 3.3. Inhibition of COX-1 and COX-2 by Wine Constituents

Thirty-three phenolic compounds (phenolic acids, flavonoids, and stilbenes) were investigated for COX-1 and COX-2 inhibitory activity to explain their impact on the overall effect of wines. Only* trans*-resveratrol strongly inhibited COX-1 and COX-2 with respective IC_50_ values 2.27 and 3.40 *μ*M (Table 3). Weak activity was recorded for quercetin (IC_50_ = 43.82 *μ*M) and kaempferol (IC_50_ ~ 60 *μ*M) in case of COX-1.

The final dilution of wine samples (200 times) in the COX assays resulted in estimated concentration of* trans*-resveratrol around 0.06 *μ*M. This concentration is calculated from the value 2.7 mg/L (11.8 *μ*M) which is the mean value of bibliographic data for content of resveratrol in red wine [25]. Evidently, even at a 10 times higher concentration of* trans*-resveratrol in wine sample could not explain the activity considering the IC_50_ values of* trans*-resveratrol. The activity of kaempferol and quercetin is negligible and no other inhibitors were identified in our screening. In accordance with the data from the literature, we propose some other compounds which could contribute to the overall effect of wine. We propose that the effect could be caused by (−)-catechin (in our study (+)-catechin was inactive at 50 *μ*M concentration) and (+)-**ε**-viniferin as Zhang et al. isolated these compounds from grape skins and claimed their COX-1 and COX-2 inhibitory activity [26]. However, they tested these compounds at high concentration 100 *μ*g/mL giving only blurred information about their real potential. Other candidates which could contribute to the activity of wine are proanthocyanidins. Garbacki et al. recorded significant inhibition of both COX forms by a gallocatechin dimer, gallocatechin-epigallocatechin dimer, and gallocatechin trimer at the 10–100 *μ*M concentrations [27]. Since the total proanthocyanidins content in red wine ranges from 250 to 2300 mg/L [28, 29], it seems that these compounds could play a more substantial role in the overall COX.

### 3.4. Inhibition of 5-LOX by Wine Constituents

Piceatannol, luteolin, quercetin, and myricetin inhibited 5-LOX with better efficiency than the reference inhibitor zileuton (IC_50_ values are stated in Table 3). However, based on the concentrations occurring in the red wines (piceatannol = 5.8 mg/L; myricetin and quercetin = 8.3 mg/L; luteolin = 1.0 mg/L; mean values of bibliographic data adopted from [25, 30]), IC_50_ values and dilution of wine samples in the 5-LOX assay (53 times) only piceatannol (estimated concentration in the assay mixture = 0.44 *μ*M; IC_50_ = 0.76 *μ*M) can contribute to the overall activity of red wines. The role of quercetin (estimated concentration = 0.52 *μ*M; IC_50_ = 3.29 *μ*M), myricetin (estimated concentration 0.34 *μ*M; IC_50_ = 4.02 *μ*M), and luteolin (estimated concentration 0.07 *μ*M; IC_50_ = 2.25 *μ*M) in the overall activity of wines seems negligible due to their low concentration in the assay mixtures. White wines are generally poor in phenolic compounds. Piceatannol, quercetin, myricetin, and luteolin are present in very low concentrations or beneath detection limits in white wines [31, 32]. Although Leifert and Abeywardena recorded the inhibition of 5-LOX (in enzymatic assay) by grape seed extract (IC_50_ = 13 *μ*g/mL) and commercial perpetration “red wine polyphenolic compounds” (IC_50_ = 35 *μ*g/mL), active constituents responsible for its activity were not identified or suggested in their study [33]. However, as in the case of COX enzymes, galloylated proanthocyanidins were able to inhibit 5-LOX activity with IC_50_ ranging from 6.6 to 18.7 *μ*M [34]. This hypothesis works for red wines, but in white wines the proanthocyanidins concentrations are almost 100 times lower [35]. Therefore, the compounds responsible for overall 5-LOX inhibitory activity especially of white wines remain unknown.

### 3.5. Docking Studies

**Figure 1:**
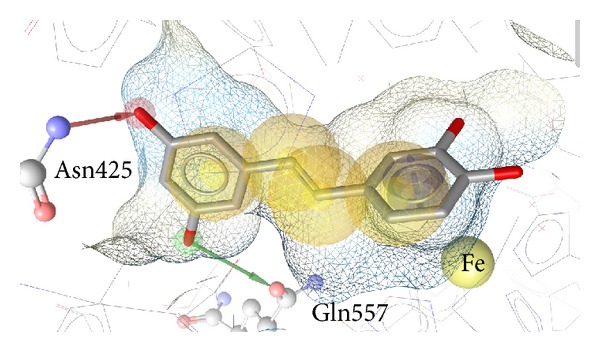
Docking pose of piceatannol in 5-LOX. Yellow spheres signify hydrophobic interactions with the binding pocket. The blue circle marks an aromatic interaction with the binding pocket. The green arrow signifies a hydrogen bond donor interaction with Gln557. The red arrow signifies a hydrogen bond acceptor interaction with Asn425.

To further elucidate the mode of inhibition of the most active compounds, they were docked into the crystal structure of 5-LOX. Piceatannol, the most potent 5-LOX inhibitor, showed several interactions with the binding pocket, most notably hydrogen bonds with Asn425 and Gln557 (Figure 1).

**Figure 2:**
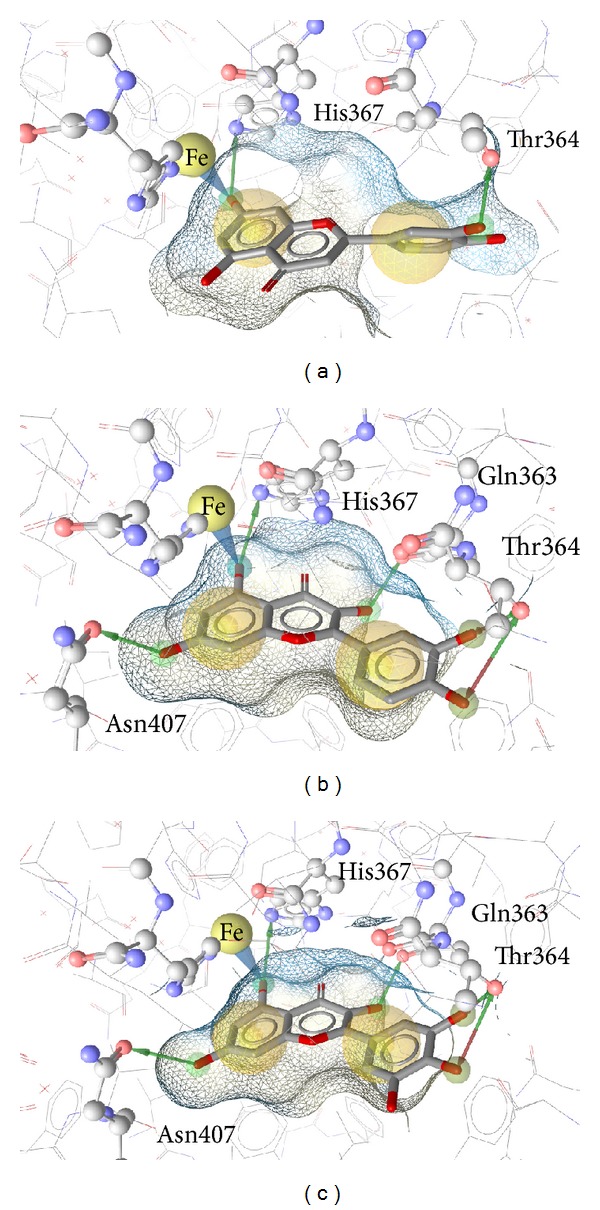
Docking poses of luteolin (a), quercetin (b), and myricetin (c). The blue cone marks a metal coordination feature formed with the iron by all three compounds. Additionally, all form hydrogen bonds with His367 and Thr364. Quercetin and myricetin also form H-bonds with Asn407 and Gln363.

Luteolin (Figure 2(a)), quercetin (Figure 2(b)), and myricetin (Figure 2(c)) all displayed a set of very similar interaction patterns. All three compounds coordinated to the catalytic iron and formed stabilizing hydrogen bonds with His367 and Thr364. Quercetin and myricetin also formed hydrogen bonds with Asn407. If a hydroxyl group on the pyrane ring (quercetin and myricetin) is present, a hydrogen bond with Gln363 is formed.

In case of COX, we recorded significant activity only for* trans*-resveratrol. Its binding pattern was already described [36, 37]. Here, it should be mentioned that Murias et al. recorded for piceatannol IC_50_ = 4.713 *μ*M and 0.0113 *μ*M for COX-1 and COX-2, respectively, resulting in a COX-2 selectivity index of 417 [5]. In contrast, no inhibition of COX-1 or COX-2 was recorded in our assays. Our results are in concordance with Lee et al. who recorded no inhibition of both COX forms by piceatannol [38] and Gerhäuser et al. who tested COX-1 inhibition with IC_50_ = 81.4 *μ*M [39]. It is difficult to explain why similar studies using enzymatic assays produced so different results. Nevertheless, it should be kept in mind that also piceatannol could be a potential COX inhibitor present in wine. A more detailed study, focusing on piceatannol should reveal the COX inhibitory potency of this interesting compound.

## 4. Conclusions

Red wines were potent inhibitors of all three tested enzymes with efficacy decreasing from COX-1 through COX-2 to 5-LOX. The evidence that red wine is a better inhibitor of COX-1 than COX-2 could contribute to its cardioprotective effect. White wines were weaker inhibitors of 5-LOX than red wines and did not inhibit COXs. The two exceptions were Georgian samples fermented with pomace (skins, stems, and seeds) by a traditional Kakhetian method. The processing method influenced inhibitory activity more than the variety of wine or its geographical origin.* Trans*-resveratrol proved to be a significant inhibitor of both COX-1 and COX-2, but the activity of this compound alone could not be responsible for overall inhibitory activity of red wines. Similarly, although piceatannol, luteolin, quercetin, and myricetin were potent inhibitors of 5-LOX, considering ratio between their IC_50_ values and their concentration in wine only piceatannol could substantially contribute to the overall activity of red wines. Since the compounds identified in our study could not fully explain the overall activities of wine, we hypothesize, based on the literature data [27, 34], that proanthocyanidins in wine could also contribute to its overall potential. However, further studies are needed for the identification of all COX-1, COX-2, and 5-LOX inhibitors contained in the wine.

## Supplementary Material

Supplementary Table provides information about variety, producer, vintage, origin (country and region), and type for each wine tested in this study.

## Figures and Tables

**Table 1:** Inhibition of COX-1, COX-2 (concentration of wine: 5 mL/L), and 5-LOX (concentration of wine: 18.87 mL/L) enzymatic activity by red wines.

Number	Variety and origin	Inhibition against blank (%)		
		COX-1	COX-2	5-LOX
1	Pinot Noir, Czech Rep.	46.13	33.25	79.96
2	Pinot Noir, Czech Rep.	90.25	29.23	77.61
3	Pinot Noir, Austria	70.05	29.20	82.74
4	Pinot noir, France	94.04	52.23	85.01
5	Pinot noir, France	85.57	22.78	84.21
6	Cabernet Sauvignon, Italy	73.52	12.21	81.92
7	Cabernet Sauvignon, Italy	87.20	23.81	83.81
8	Cabernet Sauvignon, France	92.16	37.60	80.96
9	Cabernet Sauvignon, France	92.98	43.14	82.38
10	Cabernet Sauvignon, Czech Rep.	81.42	42.14	77.59
11	Cabernet Sauvignon, Czech Rep.	48.95	34.95	71.36
12	Cabernet Sauvignon, Georgia	77.57	13.68	83.86
13	Cabernet Moravia, Czech Rep.	52.67	13.43	82.56
14	Cabernet Moravia, Czech Rep.	73.68	26.91	85.39
15	Saperavi, Georgia	90.98	25.33	84.86
16	Saperavi, Georgia	90.74	43.73	84.33
17	Saperavi, Georgia	90.31	53.24	80.65
18	Saperavi, Georgia	82.30	35.23	88.29
19	Saperavi, Georgia	38.41	−7.23	62.24
20	Saperavi, Georgia	84.09	25.42	78.68
21	Saperavi, Georgia	95.48	52.95	73.08
22	Saperavi, Georgia	95.13	63.86	80.15
23	Saperavi, Georgia	89.07	28.99	77.79
24	Saperavi + Saperavi Budeshuriseburi, Georgia	92.65	40.27	71.65
25	Saperavi + Saperavi Budeshuriseburi, Georgia	94.35	45.03	72.89
26	Alexandrouli, Georgia	82.27	22.28	79.02

Data is presented as the mean value.

**Table 2:** Inhibition of COX-1, COX-2 (concentration of wine: 50 mL/L), and 5-LOX (concentration of wine: 18.87 mL/L) enzymatic activity by white wines.

Number	Variety and origin	Inhibition against blank (%)		
		COX-1	COX-2	5-LOX
27	Chardonnay, Czech Rep.	10.91	11.94	41.74
28	Chardonnay, Czech Rep.	9.22	−7.05	47.66
29	Chardonnay, Italy	−5.48	−3.11	47.52
30	Chardonnay, Italy	3.62	4.59	47.44
31	Chardonnay, France	11.26	8.82	51.30
32	Chardonnay, France	17.24	10.95	57.99
33	Sauvignon Blanc, Italy	7.42	−21.85	51.25
34	Sauvignon Blanc, France	19.09	12.43	41.42
35	Sauvignon Blanc, Czech Rep.	15.26	9.82	32.42
36	Rkatsiteli, Georgia	14.37	−10.97	59.24
37	Rkatsiteli, Georgia	94.50	65.61	76.05
38	Rkatsiteli + Mtsvane Kakhuri + Khikhvi + Kisi, Georgia	95.97	63.71	71.93
39	Rkatsiteli + Mtsvane Kakhuri, Georgia	10.32	5.32	60.38

Data is presented as the mean value.

**Table 3:** IC_50_ values of wine constituents and reference inhibitors for COX-1, COX-2, and 5-LOX.

Compound	IC_50_ ± SD (*μ*M)		Ratio	IC_50_ ± SD (*μ*M)
	COX-1	COX-2	COX-1/COX-2	5-LOX
Resveratrol	2.27 ± 1.17	3.40 ± 0.50	0.67	—
Piceatannol	—*	—		0.76 ± 0.35
Luteolin	—	—		2.25 ± 1.75
Quercetin	—	—		3.29 ± 2.25
Myricetin	—	—		4.02 ± 2.37
Kaempferol	43.82 ± 18.81	—		—
Ibuprofen	13.14 ± 3.84	8.77 ± 2.55	1.49	nt^#^
Indomethacin	1.61 ± 0.72	10.12 ± 5.66	0.15	nt
Zileuton	nt	nt		4.71 ± 2.83

Data is presented as the mean value ± SD. *IC_50_ > 50 *μ*M concentration; ^#^not tested.
